# New Insight into the Molecular Mechanisms of the Biological Effects of DNA Minor Groove Binders

**DOI:** 10.1371/journal.pone.0025822

**Published:** 2011-10-05

**Authors:** Xinbo Zhang, Siyu Crystal Zhang, Dejun Sun, Jiang Hu, Anil Wali, Harvey Pass, Felix Fernandez-Madrid, Michael R. Harbut, Naimei Tang

**Affiliations:** 1 Internal Medicine, Wayne State University, Detroit, Michigan, United States of America; 2 Karmanos Cancer Institute, Detroit, Michigan, United States of America; 3 MedStart Program, Wayne State University, Detroit, Michigan, United States of America; 4 Research Institute of Respiratory and Critical Care Medicine, Baotou, Inner Mongolia, China; 5 Surgical Department of Central Hospital, Baotou, Inner Mongolia, China; 6 Center to Reduce Cancer Health Disparities, National Cancer Institute, Rockville, Maryland, United States of America; 7 Department of Cardiothoracic Surgery, New York University Medical Center, New York, New York, United States of America; 8 National Center for Vermiculite and Asbestos-Related Cancers, Environmental Cancer Initiative Karmanos Cancer Institute, Detroit, Michigan, United States of America; 9 Center for Occupational and Environmental Medicine, Providence Hospital, Southfield, Michigan, United States of America; Institut Pasteur, France

## Abstract

**Background:**

Bisbenzimides, or Hoechst 33258 (H258), and its derivative Hoechst 33342 (H342) are archetypal molecules for designing minor groove binders, and widely used as tools for staining DNA and analyzing side population cells. They are supravital DNA minor groove binders with AT selectivity. H342 and H258 share similar biological effects based on the similarity of their chemical structures, but also have their unique biological effects. For example, H342, but not H258, is a potent apoptotic inducer and both H342 and H258 can induce transgene overexpression in *in vitro* studies. However, the molecular mechanisms by which Hoechst dyes induce apoptosis and enhance transgene overexpression are unclear.

**Methodology/Principal Findings:**

To determine the molecular mechanisms underlying different biological effects between H342 and H258, microarray technique coupled with bioinformatics analyses and multiple other techniques has been utilized to detect differential global gene expression profiles, Hoechst dye-specific gene expression signatures, and changes in cell morphology and levels of apoptosis-associated proteins in malignant mesothelioma cells. H342-induced apoptosis occurs in a dose-dependent fashion and is associated with morphological changes, caspase-3 activation, cytochrome *c* mitochondrial translocation, and cleavage of apoptosis-associated proteins. The antagonistic effect of H258 on H342-induced apoptosis indicates a pharmacokinetic basis for the two dyes' different biological effects. Differential global gene expression profiles induced by H258 and H342 are accompanied by unique gene expression signatures determined by DNA microarray and bioinformatics software, indicating a genetic basis for their different biological effects.

**Conclusions/Significance:**

A unique gene expression signature associated with H342-induced apoptosis provides a new avenue to predict and classify the therapeutic class of minor groove binders in the drug development process. Further analysis of H258-upregulated genes of transcription regulation may identify the genes that enhance transgene overexpression in gene therapy and promote recombinant protein products in biopharmaceutical companies.

**Data Deposition:**

The microarray data reported in this article have been deposited in the Gene Expression Omnibus (GEO) database, www.ncbi.nlm.nih.gov/geo (accession no.GSE28616).

## Introduction

Many research studies have aimed to target specific sequences in DNA with the goal of designing drugs [1]. The minor groove of DNA is becoming a site of great interest due to its high sequence specific interactions with a large number of small molecules. DNA minor groove binders (MBs), one of the most widely studied class of small molecules, typically bind to AT-rich sequences of the DNA minor groove and may be divided into two functional classes: 1) compounds that can induce permanent DNA damage; 2) compounds that only interact physically with DNA and cause only reversible inhibition of DNA-dependent functions [2]. The Hoechst compounds, Hoechst 33258 (H258) [2′-(4-Hydroxyphenyl)-5-(4-methyl-1-piperazinyl)-2,5′-bi(1H-benzimidazole)] and its derivative Hoechst 33342 (H342) [2′-(4-ethoxyphenyl)-5-(4-methyl-1-piperazinyl)-2,5′-bi(1H-benzimidazole)] belong to the second functional class and are also the most studied MBs as model compounds for biochemical and biophysical studies of drugs that bind to the DNA minor groove. These MBs form strong reversible complexes preferentially at the nucleotide sequences with 4–5 adjacent AT base pairs in the minor groove of double-stranded B-DNA, where a particularly narrow groove with a floor lacking amino groups permits an optimization of van der Waals' contacts and hydrogen bonding [3], [4]. As a consequence of this DNA sequence-specific binding, drug and protein may cause mutual interference because they share a common sequence preference for DNA binding. Previous studies demonstrate that Hoechst dyes interfere with multiple DNA processing proteins such as topoisomerase I [5], [6] and II [7], DNA helicase [8], TATA box binding protein [9], [10], E2F1 [11], and replication protein A [12]. In fact, most proteins which bind sequence specifically to AT rich DNA regions have extensive contacts within the minor groove, and it is likely that inhibition of the binding of these factors to DNA by MBs is mediated by direct steric interference [13]. In addition, DNA sequence-specific binding MBs may be associated with a unique gene expression pattern or drug-specific gene expression signature since MBs only interact with minor groove regions in disassembled chromatin where transcription and/or replication are ongoing. Therefore, it is imperative to determine the Hoechst dye-specific gene expression signature to uncover potential biomarkers and Hoechst-specific signal transduction pathways for cancer therapy.

Extensive studies show that Hoechst dyes have anti-cancer activities like other MBs [2], [14]. Initial studies show that H258 possesses activity against L1210 murine leukemia [15] and several promising experiments in solid tumors have led to the use of this compound in phase I clinical trials in human [15]. However, a subsequent phase II trial against pancreatic carcinoma shows little response [16]. Further data demonstrate that H342, but not H258, is a potent apoptotic inducer of different types of cancers [17]. Even so, H342 and H258 share some similar biological effects due to their similar thermodynamic properties. For example, H342 and H258 poison topoisomerase I [5], [18], inhibit other DNA processing proteins in *in vitro* studies [9], and affect the cell cycle [19], [20]. Both dyes are found to be free radical scavengers and protect DNA against radiation-induced damage [21]. However, both dyes significantly enhance UV- and radio-induced cytotoxicity as sensitizers in human tumor cell lines [22], [23]. Intriguingly, both H258 and H342 can enhance transgene overexpression in *in vitro* studies [24], [25].

In addition to their similarities, both Hoechst dyes have uniquely different biological effects in the cell as well. H342 is several orders of magnitude more cytotoxic than H258. Large numbers of protein-DNA cross-links and DNA strand breaks can be detected in H342-, but not, H258-treated cells [5], [26], [27]. Early studies revealed that H342 causes rapid cell death once sufficient dye enters the nucleus of a cell [27]. But, our later studies show that the key difference of intracellular effects between the two dyes is that H342, but not H258, is a potent apoptotic inducer in different types of cancer cell lines [17] and species [28]. However, the molecular mechanisms by which different Hoechst dyes have diverse intracellular effects are still unclear. In chemical structure, H342 has a 4-ethyoxy substitution on the phenyl ring that its parent compound H258 does not, thus enhancing its membrane permeability. This minute modification in the chemical structure causes H342 pharmacodynamics to differ from that of H258. Thus, H258 and H342 are not only archetypal compounds for designing new MBs and studying the interaction of MBs with DNA, but also contrasting compounds for identifying the mechanisms by studying the difference of biological effects induced by the two Hoechst dyes.

The purpose of the present study is to determine the molecular mechanisms underlying different biological effects between H342 and H258. Our results show differential global gene expression profiles with individually unique gene expression signatures induced by H258 and H342. One application of these gene expression signatures is that they can be used to predict the effectiveness of new MBs derived from Hoechst dyes. In addition, genes consisting of the H342-specific gene expression signature may be invaluable targets for targeted cancer therapy. Furthermore, genes up-regulated by H258 may be utilized in transgene overexpression in gene therapy and could promote recombinant protein products in biopharmaceutical companies. Therefore, the present study has highlighted the new insights into molecular and pharmacokinetic mechanisms that cause diverse biological effects of Hoechst 33342 and Hoechst 33258, which can aid in the discovery of more advanced and efficient cancer treatments.

## Results

### Hoechst 33342 is a potent apoptosis inducer of human mesothelioma cell lines

**Figure 1:**
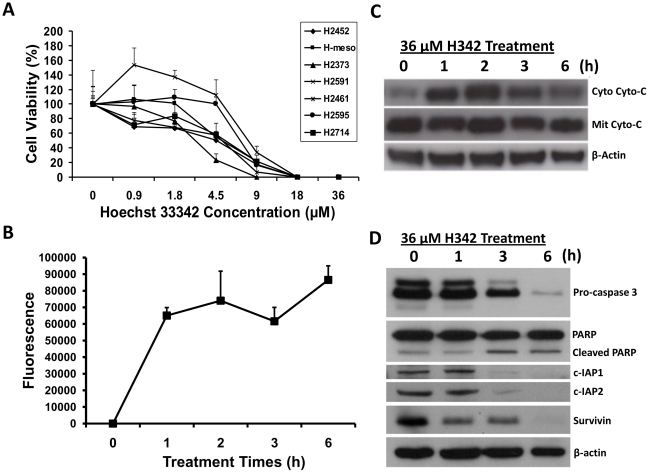
Effect of H342 on cell viability, caspase 3 activity, cytochrome *c* translocation, and apoptosis-associated protein levels. A, Effect of H342 on cell viability of 7 human mesothelioma cell lines. 7 mesothelioma cell lines were treated with different concentrations of H342 (0–36 µM) for 24 hours. Cell viability was determined by MTT assay. Line chart represents the cell viability (%) with mean + SE. B, Measurement of endogenous caspase 3 activity after 36 µM H342 for different times in H2373 cells. Line chart indicates relative fluorescence units with mean + SE. C, Determination of cytochrome *c* translocation induced by 36 µM H342 for different times in H2373 cells. After treatment, the cytosolic and the mitochondrial fractions were isolated by differential centrifugation. Levels of cytochrome *c* in cytosolic and the mitochondrial fractions were determined by immunoblotting. D, Protein levels of poly (ADP-ribose) polymerase (PARP), inhibitor of apoptosis protein 1 and 2 (c-IAP1 and 2), and caspase 3 were determined by immunoblotting after H2373 cells were treated with 36 µM H342 for different times.

**Figure 2:**
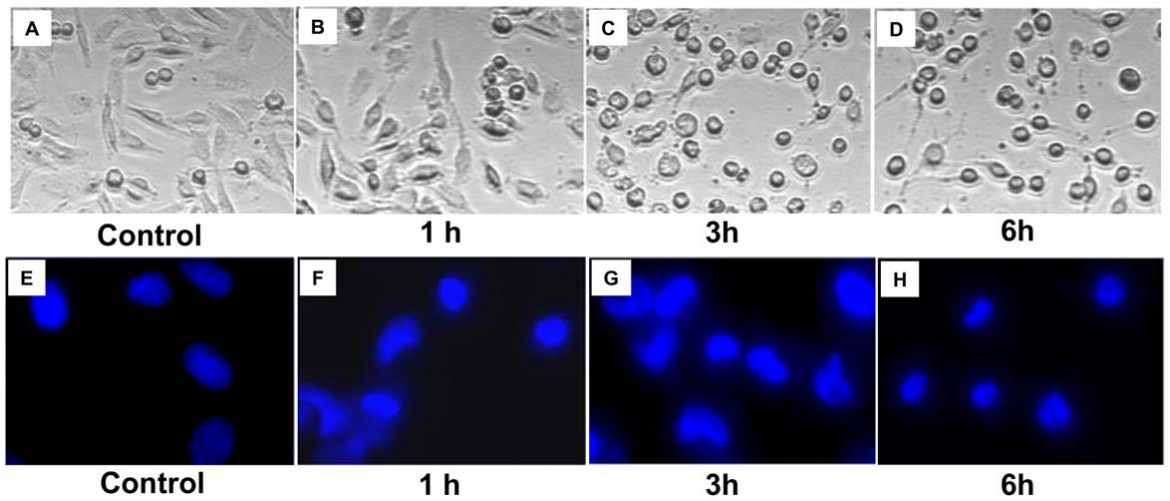
Characterization of morphological changes of H2373 cells induced by H342. A-D, The morphology of the cells was photographed with a microscope. E-H. Untreated and treated cells were rinsed with PBS buffer and stained with Hoechst 33342 (final concentration, 18 µM) for 10 minutes. After staining with Hoechst 33342, the morphological aspects of cell nuclei were observed with a fluorescence microscope.

To test whether or not H342 may trigger cell death in malignant pleural mesothelioma (MPM) cells, seven MPM cell lines have been employed in this study. Cell viability results indicate that H342 significantly induces cell death in all seven mesothelioma cell lines in dose-dependent manners by MTT (3-(4,5-Dimethylthiazol-2-yl)-2,5-diphenyltetrazolium bromide) assay (Figure 1A). To determine the nature of this death in mesothelioma cells induced by H342, we have used fluorescent staining assay, Western blot, and caspase-3 activity assay to test cell morphological changes, caspase-3 activity, and expression of apoptosis-associated proteins during the cell death of H2373 mesothelioma cells treated by H342 for different times. The cells shrink, become circular, and lose contact with neighboring cells as nuclear condensation occurs, forming half-moon shapes typical of apoptosis in a time-dependent fashion during 36 µM H342 treatment (Figure 2). The apoptotic nature of this death has been further confirmed by measurement of caspase activation, translocation of cytochrome *c*, and levels of apoptosis-associated proteins in H2373 cells treated for different intervals with 36 µM H342. Consistent with caspase 3 activation (Figure 1B), cytochrome *c* is translocated from the mitochondria to the cytosol in the response to H342 treatment (Figure 1C). Figure 1D shows that degradation of PARP, inhibition of apoptosis protein 1 (c-IAP1), c-IAP2, and survivin is associated with a decrease in pro-caspase 3 levels in H342-treated H2373 cells.

### Antagonistic effect of Hoechst 33258 on Hoechst 33342-induced apoptosis

**Figure 3:**
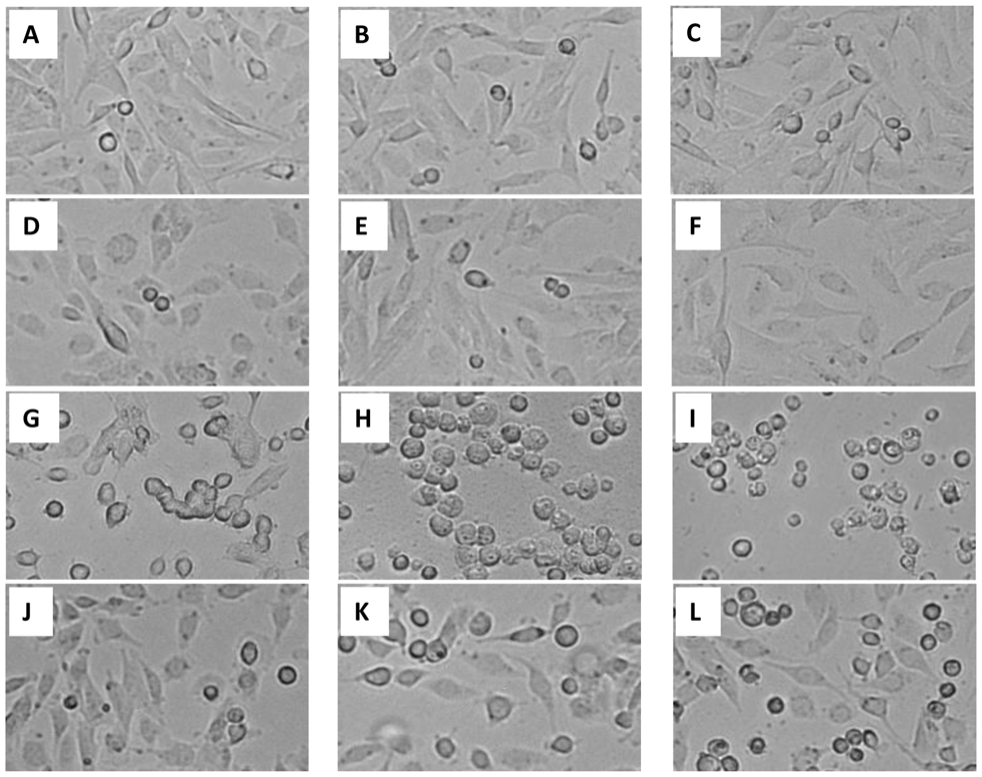
Effect of H258 on morphological changes induced by H342 in H2373 cells. H2373 mesothelioma cells were treated with H342 or H258 alone, or combination of H342 + H258 for different times. Control: A (1h), B (3h) and C (6h); 36 µM H258-treated: D (1h), E (3H), F (6h); 36 µM H342-treated: G (1h), H (3h), I (6h); 36 µM H258- and 36 µM H342-treated: J (1h), K (3h), L (6h).

**Figure 4:**
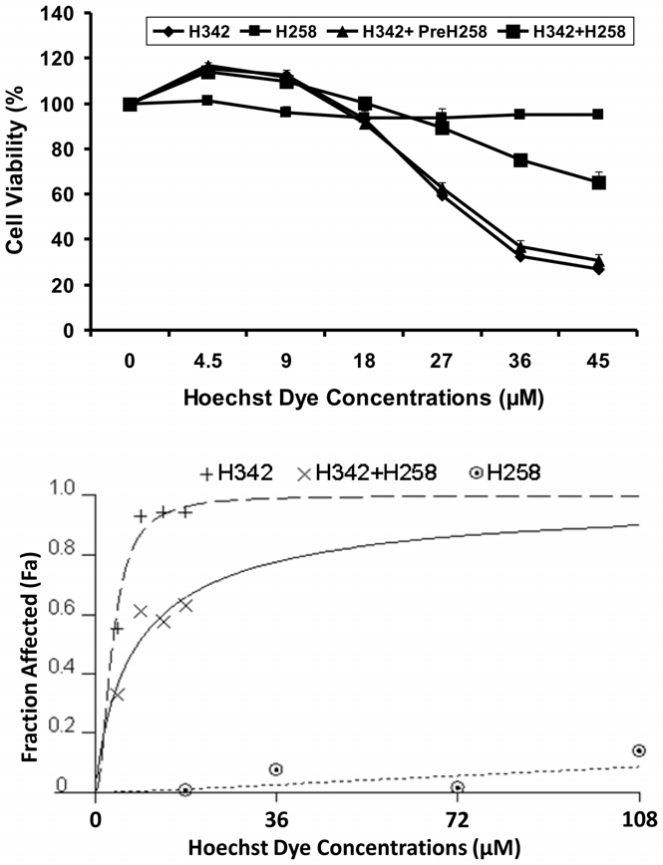
Antagonistic effect of H258 on H342-induced cell death in H2373 cells. A, Cell viability was determined by MTT assay after H2373 cells were treated by H342 or H258 alone, H258 pretreatment for 1 h followed by H342 treatment, or combination of H342 + H258 for different times. B, Antagonistic effect between H342 and H258 was analyzed by Calcusyn software to generate a dose response curve. Fa represents the fraction of cells that is growth inhibited in response to H342 and/or H258. This is calculated as 1 − fraction of surviving cells. Fa values for each treatment were used to conduct synergy analysis by CalcuSyn software as described in Materials and Methods.

To determine the pharmacokinetic relationship between H342 and H258, we postulate that H342 and H258 share the same transport system due to their similarities in chemical structure. To test this hypothesis, we examined the effects of incremental doses of H258 and H342, alone or in combination, on the cell viability of H2373 mesothelioma cells by MTT assay. Consistent with previous results, both MTT and cell morphological results show that H258 fails to induce cell death after 6 hour and 24 hour treatment at different concentrations when compared to H342-treated and H258-pre-treated groups (Figure 3 and 4A). In contrast, cell viability, as determined by MTT assay, reveals that H258 decreases H342-induced apoptosis by 33% to 57% in a dose-dependent manner (Figure 4A). Morphological data indicate combined treatment of H342 and H258 delays cell morphological changes at least for two hours when compared to H342 treatment alone or H258-pretreated group, suggesting H258 effectively delays the entry of H342 into cells (Figure 3). To further analyze drug antagonism between the two Hoechst dyes, a dose response curve is generated for Hoechst dyes in H2373 mesothelioma cells using Calcusyn software (Figure 4B). Consistent with MTT data, the combination treatment causes a significant decrease in H342-induced apoptosis when compared to that achieved in response to solely H342 (Figure 4B). The fraction of cells affected in response to each treatment is utilized to perform antagonistic analysis with Calcusyn. The CI (combination indices) as formulated by the software, revealed values of more than 1.0, indicating an antagonistic interaction between H258 and H342 when they are combined (Table 1). This analysis further confirms the drug antagonism between the two Hoechst dyes.

**Table 1:** Combination indices for H342 and H258 treatment for 24 h, as computed by CalcuSyn for H2373 cells.

H342 (µM)	Fa	H258 (µM)	Fa	H342+H258 (Fa)	CI
4.5	0.0133	18	0.0054	0.0329	1.186
9	0.3218	36	0.0231	0.1735	1.416
13.5	0.8759	72	0.0391	0.2392	1.918
18	0.9342	108	0.1133	0.3050	2.356

Note: CI (combination indices)>1 indicates antagoniusm; Fa, fraction affected.

### Differential global gene expression profiles induced by Hoechst dyes

**Figure 5:**
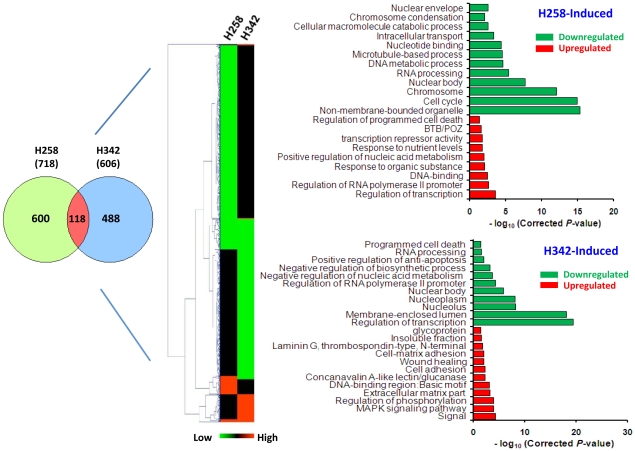
Differential gene expression profiles induced by H258 and H342. Venn diagram (left) illustrating overlap between changes identified with H342- and H258-induced differential gene expression profiles. Hierarchical cluster (middle) indicating differential gene expression induced by H258 and H342: red (up-regulated) and green (down-regulated). Gene ontology analysis (right) (*P*-values represent a Bonferroni-corrected EASE score). These genome-wide gene expression profiles were normalized by untreated H2373 cell gene expression profile.

**Figure 6:**
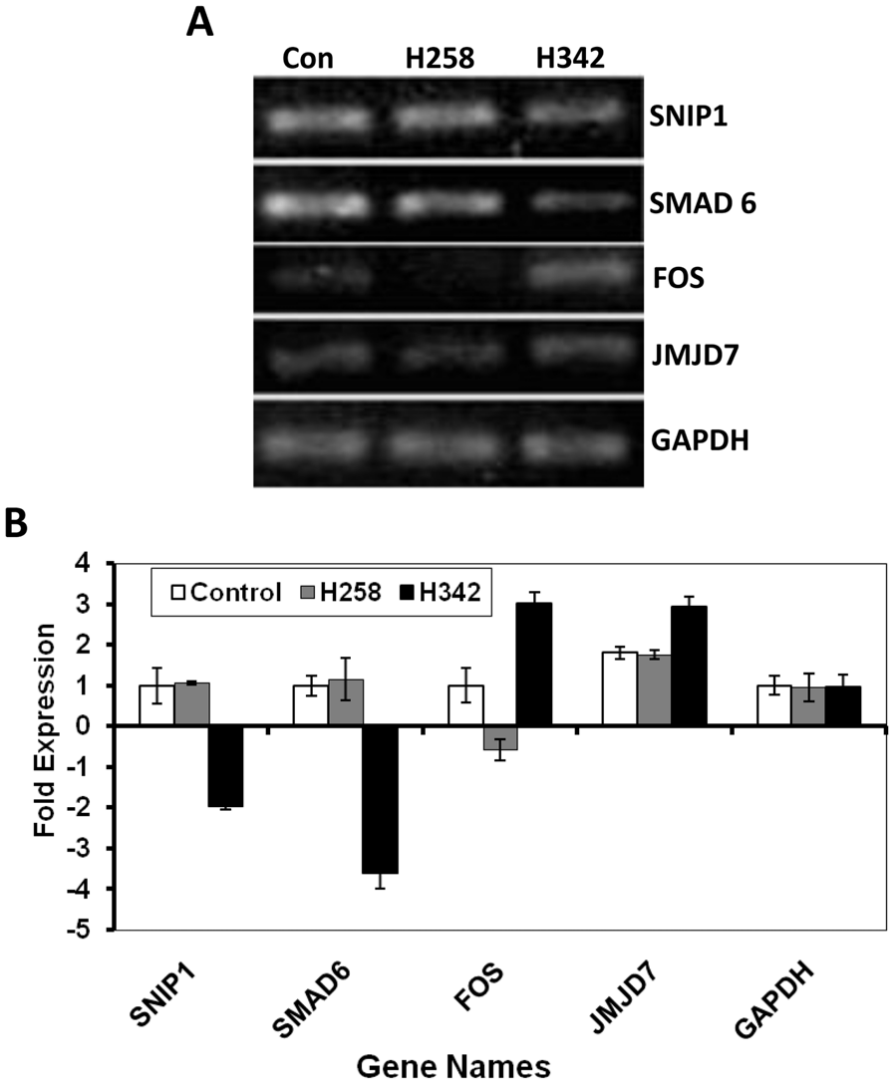
Real-time RT-PCR for microarray data validation. A, Four gene products of the real-time RT-PCR were visualized after separation on an agarose gel. B, Comparing differences of PCR cycles of four genes.

**Figure 7:**
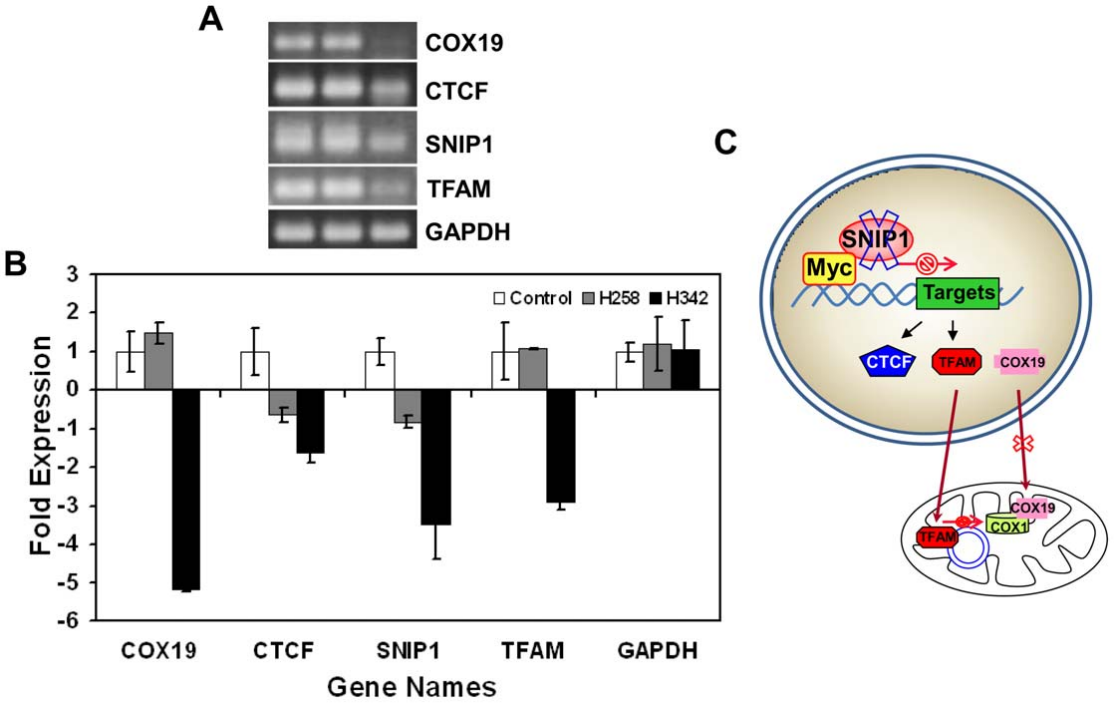
H342 downregulates SNIP1-stimulated gene expression of c-Myc target genes that are associated with mitochondrial function in H2373 MPM cells. A, Four gene products of the real-time RT-PCR were visualized after separation on an agarose gel. B, Comparing differences of PCR cycles of four genes. C, Schematic diagram of H342-induced mitochondria dysfunction through SNIP1 and COX19. In untreated H2373 MPM cells, C-terminus of nuclear protein SNIP1 interacts with the N-terminal c-Myc, resulting in enhanced transcriptional activation of c-Myc-dependent genes [30]. In addition, COX19 participates in the biogenesis mitochondrial respiratory chain complexes [35]. H342 rapidly attenuates gene expression of SNIP1 and consequently causes downregulation of c-Myc target genes such as TFAM, resulting in downregulation of gene expression of mitochondria such as COX1 [34]. COX19 downregulation fail to organize cytochrome c oxidase. Overall, these alterations of gene expression induced by H342 may lead to mitochondrial dysfunction, for good reason-H342-induced apoptosis is a mitochondria-mediated apoptosis.

Since the thermodynamic features of DNA binding of H342 and H258 are similar, the pharmacokinetic differences in the entry of H342 and H258 into cells seem to be the initial cause of the different biological effects of Hoechst dyes [29]. Since AT rich sequences of DNA are primary binding sites of Hoechst dyes, we postulate that the different biological effects of Hoechst dyes eventually stem from differential global gene expression profiles defined by their unique gene expression signatures. To test the hypothesis, we performed oligo microarray analysis of gene expression profiles in H2373 mesothelioma cells after 3 hours of 36 µM H258 or H342 treatment. Utilizing data obtained from the HumanHT-12 v4 expression BeadChip kit (Illumina), we performed hierarchical clustering using the Mev version 4.5.1 software and constructed a dendrogram for Hoechst dye-treated samples. The concordance between the results from the hierarchical cluster analysis and Venn diagram indicated that the global gene expression pattern of H342 induction was different from that of H258 induction, revealing differential gene expression profiles of cells in response to H342 and H258 treatment (Figure 5). To validate the results of our expression microarrays, we carried out quantitative RT-PCR analysis of the RNAs used in the expression microarray studies. We have analyzed a total of 4 genes: 2 genes that are up-regulated in H342-treated H2373 mesothelioma cells (Fos, JMJD7) and 2 genes that are down-regulated (SNIP1, SMAD6) (Figure 6). As shown in Figure 6A and 6B, we are able to confirm significant up- or down-regulation (P<0.05) as predicted from the microarrays. Recent study shows that SNIP1 is an important modulator of c-Myc activity [30] and c-Myc target genes encode global chromatin regulators (e.g., CTCF and hGCN5) and critical regulators of mitochondrial function such as TFAM and NRF1 [31]. Since mitochondrial dysfunction is a hallmark of H342-induced apoptosis [32]–[34] we have further chosen two c-Myc target genes (e.g., CTCF and TFAM) and COX19 that are downregulated in H342-induced gene expression profile to test the relevance of SNIP1-mediated c-Myc target genes and COX19 [35] to chromatin structure/function and mitochondrial function [31]. Figure 7A and 7B show the downregulated gene expression of CTCF, TFAM and COX19 induced by 36 µM H342 treatment for 3 hour, indicating the potential molecular basis of chromatin and mitochondria dysfunction in H342-treated cells. Figure 7C is a schematic diagram of H342-induced mitochondria dysfunction through SNIP1 and COX19. The present study represents the first large-scale and high-quality transcriptome analysis in Hoechst dye-induced gene expression.

### Two unique gene expression signatures induced by two Hoechst dyes

A gene signature consists of a list of genes whose expression is correlated with the biological state of interest. Applying highly stringent criteria, our global gene expression data have demonstrated that 99 genes are upregulated and 582 genes are downregulated after 3 hour H258 treatment, whereas 191 genes are upregulated and 490 genes are downregulated after 3 hour H342 treatment. To gain insight into the biological meaning of the Hoechst dye–specific signatures, we used David Bioinformatics Databases (http://david.abcc.ncifcrf.gov/) to examine the expression of a compendium of differential expression gene profiles representing specific ontology terms in response to H258 treatment and H342 treatment, respectively [36]. To lend biological relevance to these gene profile data, we have examined the lists of up-and down-regulated genes for over-representation of any functional classes using the DAVID Bioinformatics Database. In H258-induced gene expression profiling, nine classes were significantly over-represented among the upregulated genes of H258 treatment. These genes are mainly involved in regulation of transcription (26%), DNA binding (19%), response to organic substances and nutrients (10%), and apoptosis (10%). Five functional classes out of nine are associated with gene transcription and DNA binding (34) (Figure 5). In contrast, 582 genes are downregulated and found to be related to regulation of nuclear components (chromosomes, microtubules, the nuclear body, etc.) and nuclear biological processes (cell cycle, macromolecule metabolism and assembly, etc.) (Figure 5). Thus, it is likely that upregulation of the transcription regulation genes and downregulation of the nuclear structure and cell cycle genes are the signature of the H258-induced gene expression profile. In H342-induced gene expression profiling, twelve classes are significantly over-represented among the upregulated genes. These genes are involved in intracellular biological processes (signal, MAPK signaling pathway, cell adhesion, etc.) and cellular components such as the extracellular matrix part (Figure 5). These data suggest that cells prevent H342-induced damage by enhancing intracellular biological processes and reinforcing cellular components. Among the down-regulated genes, thirteen classes are found to be related to the regulation of nuclear metabolism, including nuclear components and nuclear biological processes (Figure 5). In contrast to the H258-downregulated gene expression profile, H342 mainly targets nuclear processes, in particular, transcription regulation (30%). Thus, it is likely that downregulation of the genes of transcription regulation is the H342-specific gene expression signature.

### Identification of signal transduction pathways consisting of these gene signatures

**Figure 8:**
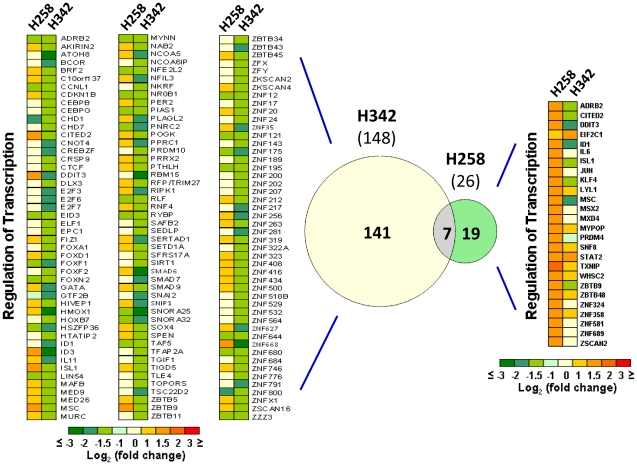
Comparison of gene expression signatures induced by H258 and H342. Heat map showing kinetics of changes in expression of transcription regulation genes identified in H342- and H258-induced global gene expression profiles based on DAVID Bioinformatics Database gene descriptions.148 H342-downregulated transcription regulation genes (left). Venn diagram (middle) illustrating 7 overlapped genes between H342-downregulated and H258-upregulated genes of transcription regulation. 26 H258-upregulated transcription regulation genes (right).

H258-upregulated and H342-downregulated genes that involve transcription regulation are the signatures of differential global gene expression profiles induced by H342 and H258. Comparing these two sets of genes shows a statistically significant overlap of only 7 genes, the majority of which is regulated in the same direction (Figure 8). To further find the enriched pathways of the two gene sets related to H342- and H258-induced transcription regulation, we performed an overrepresentation pathway analysis on Genomatix. Under the threshold of a P value of <0.05, there are 35 enriched pathways for 26 H258-upregulated genes and 10 enriched pathways for 148 H342-downregulated genes. Of the 10 enriched pathways for H342-downregulated genes (Table 2), 9, except for the vitamin D receptor, overlap with the pathways for H258-upregulated genes. Of the 7 shared genes, 5 genes (e.g. Cited2, DDIT3, Isl1, id1 and MSC) involve 8 H342-enriched pathways. These data further prove both Hoechst dyes may share the same primary targets.

**Table 2:** Pathways in which 148 H342-downregulated genes of transcription regulation involved.

Pathways	P-value	# Genes (obsreved)	# Genes (total)
NOTCH	7.72E-07	20	580
TGF BETA	6.34E-06	31	1408
RETINOBLASTOMA 1	1.98E-05	12	277
MOTHERS AGAINST DPP HOMOLOG	1.49E-04	19	758
WINGLESS TYPE	2.62E-03	19	954
PEROXISOME PROLIFERATOR ACTIVATED RECEPTOR GAMMA	2.73E-03	10	350
HISTONE DEACETYLASE	3.49E-03	8	247
TUMOR PROTEIN P53	3.95E-03	19	989
ARYL HYDROCARBON RECEPTOR	7.14E-03	6	168
VITAMIN D (1,25 DIHYDROXYVITAMIN D3) RECEPTOR	8.21E-03	5	123

## Discussion

Hoechst 33342 (H342) is a novel apoptotic inducer in different types of cancer cell lines [17] and species [28]. Conventionally, apoptosis occurs via two main pathways: the intrinsic pathway mediated by mitochondria, resulting in the activation of caspase 9, or the extrinsic pathway mediated by the activation of death receptors (Fas and TNFR), involving the activation of caspase 8 [17], [37]. Cytochrome *c* release is a crucial step in the mitochondrial or intrinsic pathway due to its ability to activate caspase 3. Since both the extrinsic and intrinsic pathways converge at caspase 3, cytochrome *c* release can be used to determine the pathway involved. Capase 3, together with other effector caspases (such as caspase 7 and 6), orchestrates the dismantling of diverse cell structures through cleavage of specific substrates [38]. Caspases, including caspase 3, exist as inactive proenzymes that undergo proteolytic processing at conserved aspartic residues to produce a cleaved active enzyme that is detected by immunoblot. A classical substrate for caspase 3 is poly ADP ribose polymerase (PARP). Our results show that degradation of PARP, inhibition of apoptosis protein 1 (c-IAP1), c-IAP2, and survivin are associated with a decrease in pro-caspase 3 levels and an increase in caspase 3 activity that are related to cytochrome *c* translocation from the mitochondria to the cytosol. Degradation of PARP impairs the DNA repair process, whereas c-IAP1, c-IAP2 and survivin downregulation disarms their antiapoptotic capacity [39], thus enhancing the apoptosis process. However, it is important to note that in this study, the signal of cleaved caspase 3 is undetected by immunoblotting. This raises the question of whether the synthetic peptide corresponding to residues that have been used for antibody generation is not included in the activated caspase 3 when H342-induced cleavage occurs, indicating the possibility of other cleavage sites for caspase 3 activation. Overall, H342-induced apoptosis is an intrinsic pathway-dependent apoptosis that is characterized by not only a morphological hallmark, but also the key biochemical features of apoptosis, including mitochondrial membrane potential [33], externalization of membrane phophoatidylserine [40], DNA fragmentation [40], [41], activation of caspase 3 associated with cytochrome *c* release (Figure 1C) [42], and cleaved caspase 3 substrates (Figure 1D). Moreover, H342-induced apoptosis is also associated with dysfunctions of Lactate oxidation [32] and fatty acid anabolism [43]. Thus, H342-induced apoptosis is complicated and still remains unclear.

Malignant pleural mesothelioma (MPM) is a deadly and asbestos-associated disease with patient survival from presentation of <12 months [44]. Unfortunately, no single therapy has proven effective in curing MPM, presumably because of the multiplicity of survival and chemoresistance pathways in these tumors [45]. Substantial improvements in survival will require the development of novel and more effective pharmacological interventions. In addition, nearly all MPM patients progress during or after first-line treatment, and second-line chemotherapy is being increasingly used in clinical practice. Therefore, second-line therapy of MPM remains an ideal field in which to test new chemotherapy agents as well as new therapeutic strategies, including anti-angiogenic compounds, small molecules or monoclonal antibodies that target different molecular pathways [46]. In the present study, H342 acts as a potent apoptotic inducer in mesothelioma cell lines, which indicates that MBs may hold promise as a novel therapeutic approach for MPM treatment.

Hoechst dyes are utilized for staining DNA to evaluate the cell cycle and apoptosis, and quantify viable cells in molecular biology [17]. H342 efflux technique has been widely used for identifying side population cells from a variety of tissues and species, including cancer cell lines and tissues. The efflux ability of the cells has been attributed to the high expression of the ATP binding cassette (ABC) transporter G2 and multidrug resistance protein 1 [47]. However, the mechanism by which a cell readily takes up Hoechst dyes remains unclear. The present results show that pretreatment of H258 for one hour fails to protect H2373 cells from H342-induced apoptosis, and H342 approaches its maximum peak emission spectra for the formation of H342-DNA complexes in spermatozoa after a 30 minute incubation period [48], thus indicating the antagonistic effect of H258 on H342-induced apoptosis is not associated with pre-occupied AT-rich DNA sequences by H258 (Figure 3A). However, since H342-induced apoptosis is partially blocked by H258, this implies that Hoechst dyes at least partially share the same transport system. Previous literature has suggested that H342 enters the cell by an unmediated diffusion transport mechanism through the cell membrane prior to DNA binding [48], [49]. In addition, our initial data shows that the components of the cell culture media affect the potency of H342-induced apoptosis [41]. Antagonism between Hoechst dyes and among Hoechst dyes and other components of the media may imply that there is existence of a mediated transport mechanism involving Hoechst uptake. Therefore, further experiments are required to determine whether or not Hoechst dyes pass through the cell membrane by not only simple diffusion, but also by mediated transport mechanisms. Our present result suggests that the pharmacokinetic reason for the two Hoechst dyes' diverse biological effects on the cell is due to differences in cell membrane transport of the two dyes into the cell.

In the present study, our gene expression profiles in response to the treatment of the two Hoechst dyes show differential global gene expression profiles with unique gene expression signatures. It is likely that upregulation of the transcription regulation genes and downregulation of the nuclear structure and cell cycle genes are the signature of the H258-induced gene expression profile. This signature discloses the molecular mechanisms behind previous findings: inhibition of constitutive heterochromatin condensation [50], prolongation of the G2 cycle [51], and enhancement of transgene overexpression [25]. In contrast to the H258 signature, it is likely downregulation of the genes involved in transcription regulation is the H342-specific gene expression signature. Consistent with H342-response gene expression signature, H342-induced apoptosis is not related to *de novo* synthesis of RNA and proteins [32], which is associated with rapid degradation of multiple critical proteins such as replication protein A [12], TATA box binding protein [10], fatty acid synthase [43]. There are approximately 2600 proteins in the human genome that contain DNA-binding domains, and most of these are presumed to function as transcription factors [52]. Thus, only a paucity of transcription regulation genes (less than 7% of the total transcription regulation genes) are affected by Hoechst dyes, indicating that Hoechst dyes are highly specific DNA binders. Of the 10 enriched pathways for 148 H342-downregulated genes of transcription regulation, 9 (except for the vitamin D receptor) overlap with the pathways for H258-upregulated genes of transcription regulation in enriched pathway analysis of the signatures of these two dyes, demonstrating their sharing similar primary targets. Since H342-downregulated genes of transcription regulation involve some critical pathways for cell survival and development such as Notch and TGF-β, etc (Tables 2) H342 like other DNA minor groove binders may be unnecessarily over-targeting many vital genes in the cells, which results in extreme intolerance [14]. Therefore, choosing some pathways targeted by H342 for pathway-targeted cancer therapy may be a way to minimize cytotoxicity and maximize therapeutic effectiveness of DNA minor groove binders. It is worth mentioning that several expected targets, such as helicase and topoisomerase I and II, are not detected by microarray analysis, suggesting that helicase and topoisomerases may not be the initial targets of Hoechst dyes. The gene expression profile induced by camptothecin, a typical inhibitor of topoisomerase I, is characterized by downregulated genes involved in DNA metabolism and mitosis, and upregulated genes related to DNA damage stimulus and cell cycle arrest [53]. These differential gene expression profiles induced by camptothecin and the two Hoechst dyes demonstrate that drug-induced gene signatures may provide valuable information for drug reclassification, efficacy prediction, and toxicity evaluation.

## Materials and Methods

### MPM cell lines and Reagents

Seven cell lines (H2373, H2452, H2596, H2461, H2591, H-meso, and H2714) derived from patients with malignant plural mesothelioma were cultured in RPMI 1640 (HyClone) supplemented with 100 units/ml of penicillin, 100 µg/ml streptomycin, and 10% fetal calf serum (HyClone) [54], [55], [56]. Anti-PARP antibody was purchased from Biomed. Anti-caspase-3 antibody was purchased from Cell Signaling Technology and anti-actin antibody was from Sigma. Antibodies against survivin, c-IAP1, and c-IAP2 were from Santa Cruz Biotechnology, Inc. Anti-cytochome c antibody was from BD Bioscience. Fluorogenic caspase-3 specific substrate Ac-Asp-Glu-Val-Asp-AMC was from EMD Chemicals.

### MTT Assay

Determination of cell viability was studied using the MTT assay. Cells were seeded in a 96-well culture plate and subsequently treated with indicated agents for different times. After treatment, the cells were incubated with 0.5 mg/ml MTT reagent (Sigma) at 37°C for 3 h and then MTT medium was removed and 100 µL of DMSO added, followed by colorimetric analysis using a Victor3 Multilabel Plate Reader at 570 nm (Perkin Elmer). Results were plotted as the mean from triplicate experiments.

### Analysis of interaction between H342 and H258

Combination index (CI) method adapted for *in vitro* drug testing was used to determine the nature of interaction between the two agents as described previously [57]. CI was generated from MTT data by CalcuSyn software (Biosoft). Based on CI values, the extent of synergism/antagonism may be determined. In general, CI<1 suggests synergy, whereas CI >1 indicates antagonism between the drugs.

### Morphology Studies and Hoechst 33342 Staining

The treated and untreated H2373 cells were washed twice in PBS and fixed in PBS containing 1% paraformaldehyde. The morphology of the cells was then studied and photographed with a microscope.

Hoechst 33342 staining was used to observe the nuclear morphology of the cells through fluorescence microscope using a 320 to 350 nm filter. Untreated and treated cells were rinsed with PBS buffer and stained with Hoechst 33342 (final concentration, 18 µM) for 10 minutes. After staining with Hoechst 33342, the morphological aspects of cell nuclei were observed with a fluorescence microscope.

### Caspase-3 activity assay

Intracellular caspase-3 activities in cell extracts were determined by measuring the release of the AMC groups from a caspase-3 specific substrate Ac-Asp-Glu-Val-Asp-AMC. The treated and untreated cells were harvested. Cell extracts were prepared using RIPA buffer (50 mM Tris, pH 8.0, 150 mM NaCl, 1.0% IGEPAL CA-630, 0.5% sodium deoxycholate, 0.1% SDS) with protease inhibitor cocktail (Sigma). The cell extract (30 µg) was then incubated in 100 µl of the reaction buffer (50 mM Tris–HCl, pH 7.5) along with 40 µM of caspase-3 substrate in a 96-well plate. The reaction mixture was incubated at room temperature for 10 min and the hydrolyzed fluorescent AMC groups were measured using a Victor 3 Multilabel Counter with an excitation filter of 380 nm and an emission filter of 460 nm (Perkin Elmer).

### Mitochondria isolation and cytochrome *c* release assays

Isolation of mitochondria and cytochrome *c* release assays were performed by differential centrifugation as described previously [58]. In brief, untreated and treated cells were homogenized in HM buffer (10 mM HEPES, pH 7.4, 250 mM mannitol, 10 mM KCl, 5 mM MgCl_2_, 1 mM EGTA) containing 1 mM phenylmethylsulfonyl fluoride and protease inhibitor cocktail (Sigma). The homogenate was centrifuged twice at 1000 × *g* for 5 min to remove nuclei and debris, and the resulting supernatant was centrifuged at 10,000×*g* for 10 min to sediment the low speed fraction containing mitochondria. The mitochondria were washed twice with the HM buffer and resuspended in RIPA buffer with protease inhibitor cocktial (Sigma). The purity of cytosolic and mitochondrial fractions is determined by flow cytometry when the two fractions are stained by JC1, a specific mitochondrial indicator (Figure S1) [33]. For detection of cytochrome *c* release, cytochrome *c* concentrations in the cytosol and the mitochondria were determined by immunoblotting with anti-cytochrome *c* antibody (BD Pharmingen) as described below.

### Measurement of apoptosis-associated proteins by Immunoblotting

Proteins (30 µg) from either total cell lysates or cytosolic and mitochondrial fractions of untreated and H342-treated H2373 cells were separated by 12% SDS-PAGE and then transferred onto PVDF membranes. The membrane was incubated with antibodies against the following proteins: PARP, c-IAP1, c-IAP2, survivin, caspase 3, cytochrome *c* and β-actin. Secondary antibodies conjugated with horseradish peroxidase were visualized with enhanced-chemiluminescence substrates (Pierce).

### RNA extraction

Total RNA from the untreated and treated H2373 mesothelioma cells was extracted using RiboPure™ Kit (Ambion), according to the manufacturer's instruction manual.

### Microarray Expression Profiling

Microarray expression profiling was performed by the Applied Genomics Technology Center (Wayne State University). The RNA was amplified into cRNA and biotinylated by *in vitro* transcription using the Illumina® TotalPrep RNA Amplification Kit (Ambion) according to the manufacturer's protocol. Biotinylated cRNAs were purified, fragmented, and subsequently hybridized to an Illumina Human-12 v4 Expression BeadChip (Illumina). Microarray data have been submitted to the Gene Expression Omnibus (GEO) database (accession no.GSE28616). All data is MIAME compliant.

### Global gene expression analysis and signal pathway analysis

Functional interpretation of differentially expressed genes was analyzed in the context of gene ontology using the DAVID Bioinformatics Resources 6.7 (http://david.abcc.ncifcrf.gov/). The differentially expressed genes induced by two Hoechst dyes were categorized, compared to genetic categories in the David database, and ranked according to p-values [36]. Hierarchical clusters of global gene expression profiles were analyzed by the Mev version 4.5.1software and enriched pathways of gene expression signatures were analyzed by Genomatix software.

### Real-time RT-PCR for microarray data validation

To validate microarray data, 50 ng of total RNA was reverse transcribed using MMLV reverse transcriptase enzyme (Promega) in the presence of the RNase inhibitor RNAsin (2 units/µl) (Promega). Real-time PCR amplification was performed with the ABsolute QPCR SYBR Green Mix (Thermo Scientific) on a MJ Research DNA Engine Opticon (MJ Research). The initial denaturation step was at 95°C for 5 min, followed by 40 cycles of amplification at 95°C for 30s, 60°C for 30s and 72°C for 30s. PCR products were separated on a 2% agarose gel and stained with ethidium bromide. The primers used in the real-time PCRs are shown in additional file1: Table S1.

### Statistical analysis

Statistical differences between treatment groups were measured using a Student's t-test. P-values <0.05 were considered significant.

## Supporting Information

Figure S1 Determination of the amounts of the mitochondria in different fractions after fractionating the mitochondria from cultured H2373 MPM cells. The mitochondria of H2373 cells were extracted using the centrifuge-based method [58]. A, Mitochondrial fraction without JC-1 staining (0.2% mitochondria or UL+UR areas); B, Supernatant after first centrifuge (55.29%); C, Cytosolic fraction (15%) after second centrifuge; D, Mitochondrial fraction (99.11%) after second centrifuge. Since protein abundance is higher in the cytosolic fraction than that in the mitochondrial fraction, the ratio of mitochondrial amounts between the two fractions is about 500-1000 times according to the protein amount that you load for the SDS-PAGE gel.
(TIF)

Table S1 Primer sequences used for real-time RT-PCR in microarray validation analysis.
(DOC)
