# Supplementary figures and images for: New Insight into the Molecular Mechanisms of the Biological Effects of DNA Minor Groove Binders
Source: PLoS One. 2011 Oct 5;6(10):e25822. doi: 10.1371/journal.pone.0025822 (PMC3187808; doi:10.1371/journal.pone.0025822)

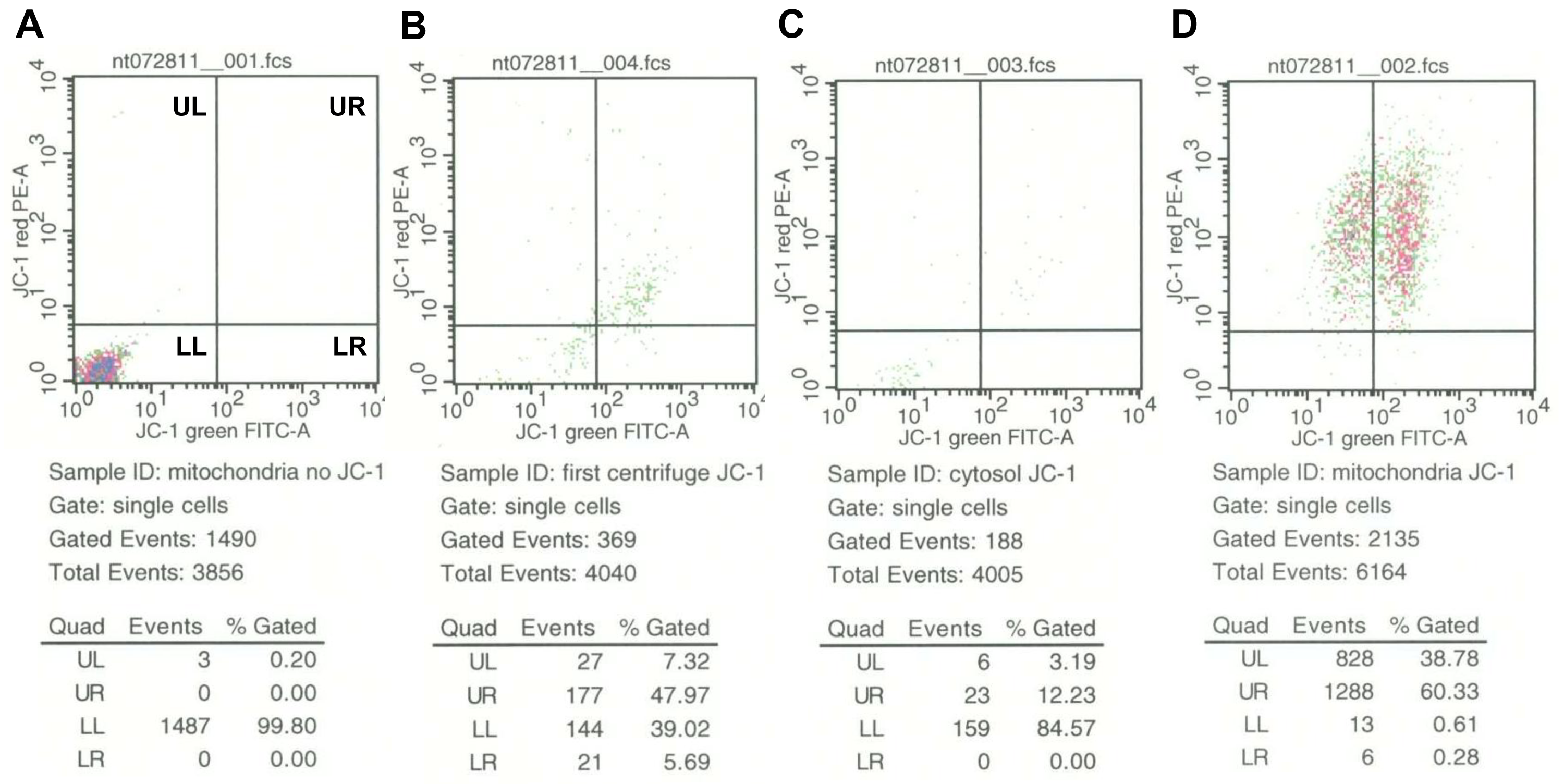

Supplement: Figure S1 — Determination of the amounts of the mitochondria in different fractions after fractionating the mitochondria from cultured H2373 MPM cells. The mitochondria of H2373 cells were extracted using the centrifuge-based method [58]. A, Mitochondrial fraction without JC-1 staining (0.2% mitochondria or UL+UR areas); B, Supernatant after first centrifuge (55.29%); C, Cytosolic fraction (15%) after second centrifuge; D, Mitochondrial fraction (99.11%) after second centrifuge. Since protein abundance is higher in the cytosolic fraction than that in the mitochondrial fraction, the ratio of mitochondrial amounts between the two fractions is about 500-1000 times according to the protein amount that you load for the SDS-PAGE gel. (TIF) [file pone.0025822.s001.tif]
