# Supplementary material for: New Insight into the Molecular Mechanisms of the Biological Effects of DNA Minor Groove Binders
Source: PLoS One. 2011 Oct 5;6(10):e25822. doi: 10.1371/journal.pone.0025822 (PMC3187808; doi:10.1371/journal.pone.0025822)
Supplement: Table S1 — Primer sequences used for real-time RT-PCR in microarray validation analysis. (DOC) [file pone.0025822.s002.doc]

**Table S1. Primer sequences used for real-time RT-PCR in microarray validation analysis**.

| **Gene Name** | **Forward Primer Sequence** | **Reverse Primer Sequence** |
| --- | --- | --- |
| JMJD7 | 5’GGTCCCTCCCCTATTTCAGA3’ | 5’TAGAGGACTCCAGGGTGCTG3’ |
| FOS | 5’CCAACCTGCTGAAGGAGAAG3’ | 5’TCAGGGTCATTGAGGAGAGG3’ |
| SNIP1 | 5’AAGGCCTCAGGGAGAAGAAG3’ | 5’CCGGTCACTGTTCCTAGCTC3’ |
| SMAD6 | 5’GGATCTGTCCGATTCCACAT3’ | 5’CTGCCCTGAGGTAGGTCGTA3’ |
| CTCF | 5’CGCCAGTTAGAAGTCAGCA3’ | 5’GTGTCCCTGCTGGCATAACT3’ |
| TFAM | 5’TGGCAAGTTGTCCAAAGAAA3’ | 5’ACGCTGGGCAATTCTTCTAA3’ |
| COX19 | 5’CTTCCCGCTGGATCACTTAG3’ | 5’CCAAATCCCAGTTTCTCCAA3’ |
| GAPDH | 5’TCACCAGGGCTGCTTTTAAC3’ | 5’GACAAGCTTCCCGTTCTCAG3’ |
